# Comorbidity of Novel *CRHR2* Gene Variants in Type 2 Diabetes and Depression

**DOI:** 10.3390/ijms23179819

**Published:** 2022-08-29

**Authors:** Mutaz Amin, Jurg Ott, Derek Gordon, Rongling Wu, Teodor T. Postolache, Michael Vergare, Claudia Gragnoli

**Affiliations:** 1 Institut National de la Santé et de la Recherche Médicale (INSERM), US14-Orphanet, 75014 Paris, France; 2 Department of Biochemistry and Molecular Biology, Faculty of Medicine, Al-Neelain University, Khartoum 11121, Sudan; 3 Laboratory of Statistical Genetics, Rockefeller University, New York, NY 10065, USA; 4 Department of Genetics, Rutgers University, Piscataway, NJ 08854, USA; 5 Department of Public Health Sciences, Penn State College of Medicine, Hershey, PA 17033, USA; 6 Departments of Statistics, Penn State College of Medicine, Hershey, PA 17033, USA; 7 Mood and Anxiety Program, Department of Psychiatry, University of Maryland School of Medicine, Baltimore, MD 21201, USA; 8 Rocky Mountain Mental Illness Research Education and Clinical Center (MIRECC), Veterans Integrated Service Network (VISN) 19, Denver, CO 80246, USA; 9 Military and Veteran Microbiome: Consortium for Research and Education (MVM-CoRE), Aurora, CO 80246, USA; 10 Mental Illness Research Education and Clinical Center (MIRECC), Veterans Integrated Service Network (VISN) 5, VA Capitol Health Care Network, Baltimore, MD 21090, USA; 11 Department of Psychiatry and Human Behavior, Sidney Kimmel Medical College, Thomas Jefferson University, Philadelphia, PA 19107, USA; 12 Division of Endocrinology, Department of Medicine, Sidney Kimmel Medical College, Thomas Jefferson University, Philadelphia, PA 19107, USA; 13 Division of Endocrinology, Department of Medicine, Creighton University School of Medicine, Omaha, NE 68124, USA; 14 Molecular Biology Laboratory, Bios Biotech Multi-Diagnostic Health Center, 00197 Rome, Italy

**Keywords:** major depressive disorder (MDD), type 2 diabetes (T2D), corticotropin-releasing hormone receptor 2 (*CRHR2*), cortisol, stress, hypothalamic–pituitary–adrenal (HPA), linkage, linkage disequilibrium (LD), association

## Abstract

The corticotropin-releasing hormone receptor 2 (*CRHR2*) gene encodes CRHR2, contributing to the hypothalamic–pituitary–adrenal stress response and to hyperglycemia and insulin resistance. *CRHR2−/−* mice are hypersensitive to stress, and the *CRHR2* locus has been linked to type 2 diabetes and depression. While *CRHR2* variants confer risk for mood disorders, MDD, and type 2 diabetes, they have not been investigated in familial T2D and MDD. In 212 Italian families with type 2 diabetes and depression, we tested 17 *CRHR2* single nucleotide polymorphisms (SNPs), using two-point parametric-linkage and linkage-disequilibrium (i.e., association) analysis (models: dominant-complete-penetrance-D1, dominant-incomplete-penetrance-D2, recessive-complete-penetrance-R1, recessive-incomplete-penetrance-R2). We detected novel linkage/linkage-disequilibrium/association to/with depression (3 SNPs/D1, 2 SNPs/D2, 3 SNPs/R1, 3 SNPs/R2) and type 2 diabetes (3 SNPs/D1, 2 SNPs/D2, 2 SNPs/R1, 1 SNP/R2). All detected risk variants are novel. Two depression-risk variants within one linkage-disequilibrium block replicate each other. Two independent novel SNPs were comorbid while the most significant conferred either depression- or type 2 diabetes-risk. Although the families were primarily ascertained for type 2 diabetes, depression-risk variants showed higher significance than type 2 diabetes-risk variants, implying *CRHR2* has a stronger role in depression-risk than type 2 diabetes-risk. In silico analysis predicted variants’ dysfunction. *CRHR2* is for the first time linked to/in linkage-disequilibrium/association with depression-type 2 diabetes comorbidity and may underlie the shared genetic pathogenesis via pleiotropy.

## 1. Introduction

The hypothalamic–pituitary–adrenal axis (HPA) plays very important roles in humans’ physiologic response in stressful conditions [1]. The HPA is activated by the release of corticotropin-releasing hormone (CRH) from the hypothalamus, which leads to the secretion of adrenocorticotropin-releasing hormone (ACTH) from the pituitary gland [2]. The secretion of ACTH stimulates the production of cortisol from the adrenal glands, which then acts as a multisystemic stress signal [3]. Cortisol release is associated with insulin resistance, hyperglycemia, and type 2 diabetes (T2D) [4]. Chronic elevation in cortisol level can lead to structural changes in the brain associated with major depressive disorder (MDD) and anxiety [5]. Studies have shown that these physiopathological changes indicate mood disorders (i.e., MDD), and insulin resistance (i.e., T2D) can occur concomitantly [6,7,8,9]. The comorbidity of T2D and MDD [10,11,12,13] can be explained by environmental, iatrogenic (e.g., antipsychotics), hormonal, or genetic factors [14]. However, MDD per se confers 60% increased risk for T2D in drug-naïve patients [6]; thus, an underlying genetic comorbidity may link the two disorders, at least in a subset of patients.

CRH effects start by binding to one of its receptors. Currently, two main CRH receptors have been identified: CRHR1 and CRHR2. Both receptors are expressed in the brain and adrenal gland, but CRHR2 is more widely distributed, with expression in the pancreas, skeletal muscles, and adipose tissue [15]. Considering the important role that the HPA axis plays in stress response and glucose metabolism, variants in *CRHR1* and *CRHR2* genes may lead to abnormal psychological and/or T2D traits and account at least in part for the comorbidity of T2D and MDD [16]. *CRHR2−/−* mice were found to have anxiety-like behaviors and be hypersensitive to stress [17], and the *CRHR2* locus in humans (7p21-p15) has been linked to T2D and MDD [18,19,20]. Risk variants in the *CRHR2* gene have been reported in patients with MDD [21], bipolar disorder [22], and post-traumatic stress disorder (PTSD) [23]; and one risk variant has been reported in T2D [24]; however, variants in comorbid MDD-T2D patients have not been studied. Considering the pleiotropic role of *CRHR2* gene in both mental and metabolic disorders, we hypothesized that risk variants in *CRHR2* gene can predispose to MDD-T2D co-morbidity. Thus, we aimed to investigate in families *CRHR2-*variants linkage and/or linkage disequilibrium (LD, i.e., association) with/to MDD and/or T2D pathogenesis, and the potential *CRHR2*-variants contribution to the genetic comorbidity of MDD and T2D.

## 2. Results

### 2.1. Linkage, Linkage Disequilibrium (LD, i.e., Association) Analysis, and LD among Single Nucleotide Polymorphisms (SNPs)

**Figure 1:**
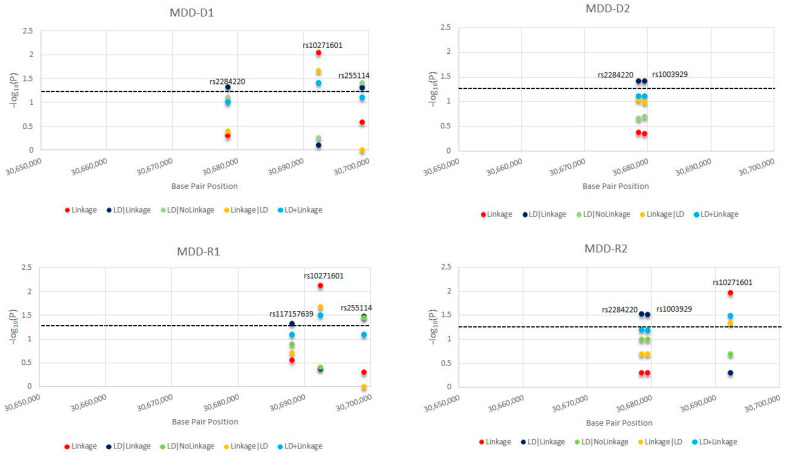
Major depressive disorder (MDD) *CRHR2*-Risk Single Nucleotide Polymorphisms (SNPs). For each *CRHR2*-risk SNPs in MDD, we present the −log_10_(P) as a function of each test statistic [Linkage, linkage disequilibrium (LD)|Linkage, LD|NoLinkage, Linkage|LD, and LD + Linkage] and per inheritance model: D1: dominant, complete penetrance, D2: dominant, incomplete penetrance, R1: recessive, complete penetrance, R2: recessive, incomplete penetrance.

**Figure 2:**
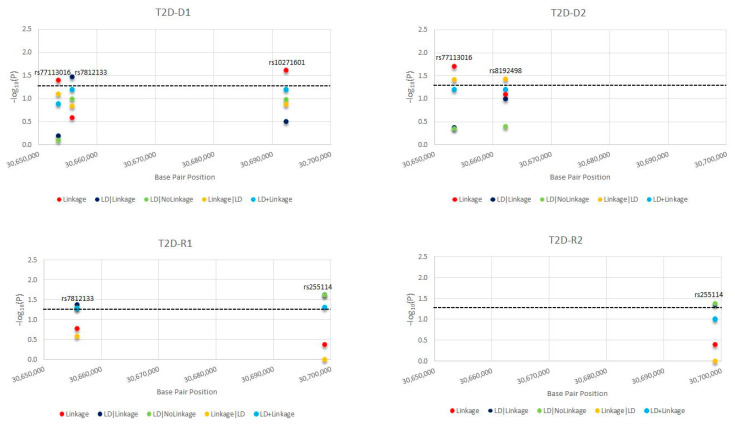
Type 2 Diabetes (T2D) *CRHR2*-Risk Single Nucleotide Polymorphisms (SNPs). For each *CRHR2*-risk SNPs in T2D, we present the −log_10_(P) as a function of each test statistic [Linkage, linkage disequilibrium (LD)|Linkage, LD|NoLinkage, Linkage|LD, and LD + Linkage] and per inheritance model: D1: dominant, complete penetrance, D2: dominant, incomplete penetrance, R1: recessive, complete penetrance, R2: recessive, incomplete penetrance.

**Figure 3:**
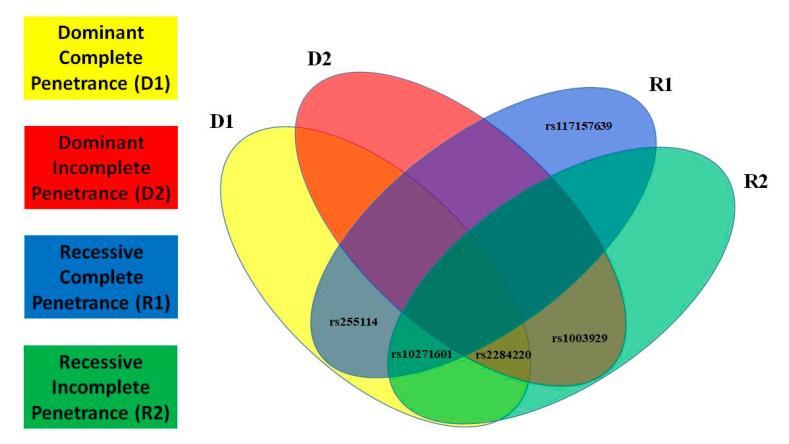
Major Depressive Disorder (MDD) Overlapping Risk Single Nucleotide Polymorphisms (SNPs). Overlapping models for *CRHR2*-risk SNPs in MDD using a Venn diagram.

**Figure 4:**
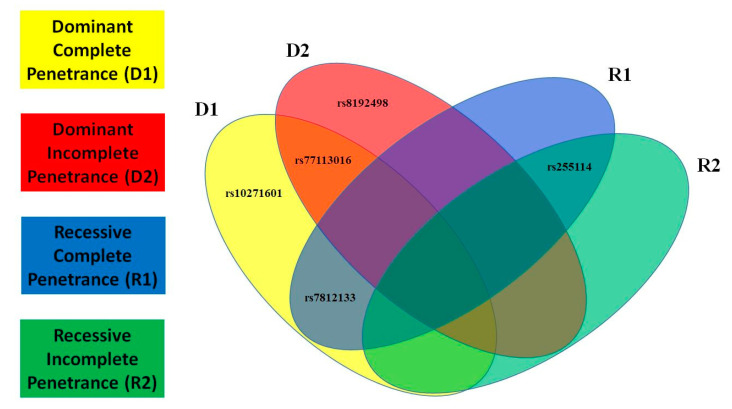
Type 2 Diabetes (T2D) Overlapping Risk Single Nucleotide Polymorphisms (SNPs). Overlapping models for *CRHR2*-risk SNPs in T2D using a Venn diagram.

In this paragraph, we present the most significant SNPs of linkage/LD tests for MDD and T2D; the statistical results of all tests and models for MDD are presented in Figure 1 and for T2D in Figure 2. We detected novel significant (*p* ≤ 0.05) linkage to and/or LD/association with MDD for: 3 SNPs/D1 (rs2284220, within LD-block Set 01, rs10271601, and rs255114); 2 SNPs/D2 (rs2284220 and rs1003929, both within LD-block Set 01); 3 SNPs/R1 (rs117157639, rs10271601, and rs255114); and 3 SNPs/R2 (rs2284220 and rs1003929, both within LD-block Set 01, and rs10271601). The MDD risk variants are all novel. We detected novel significant (*p* ≤ 0.05) linkage to and/or LD/association with T2D for: 3 SNPs/D1 (rs77113016, rs7812133, and rs10271601); 2 SNPs/D2 (rs77113016 and rs8192498); 2 SNPs/R1 (rs7812133 and rs255114); and 1 SNP/R2 (rs255114). The T2D risk variants are all novel. Two independent SNPs (rs10271601, rs255114) were comorbid and novel. The MDD-risk SNPs were in an MDD-specific LD block (Set01 containing 2 risk variants) or independent, and the T2D-risk SNPs were all independent. Specifics of the significant SNPs are provided in Table 1. The *CRHR2*-risk SNPs in MDD and T2D overlapping the parametric models are illustrated in Figure 3 and Figure 4.

### 2.2. In-Silico Functional Predictions

**Figure 5:**
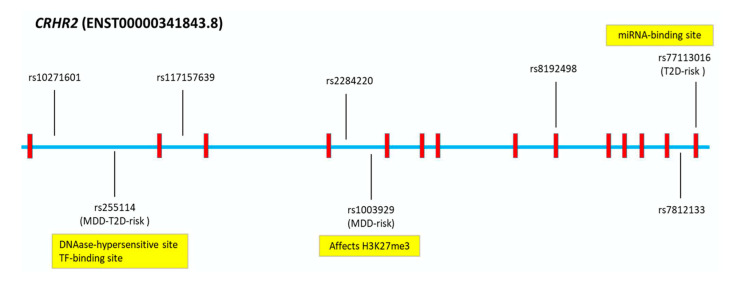
Major Depressive Disorder (MDD) and/or Type 2 Diabetes (T2D) *CRHR2*-Single Nucleotide Polymorphisms (SNPs) and In Silico Analysis. In silico functional predictions of *CRHR2*-risk variants for MDD and/or T2D.

For pathogenicity predictions, the coding variants were analyzed using Sorting Intolerant From Tolerant (SIFT) [26], Polymorphism Phenotyping v2 [27] (PolyPhen-2), and MutationTaster [28] tools. The non-coding variants were analysed using tools that predict splicing (SpliceAI) [29], transcription-factor (TF) binding (SNPnexus) [30], SNP Function Prediction [31], regulatory potential (RegulomeDB) [32], and miRNA binding [33]. The non-risk allele (A) of the coding SNP rs77113016 (c.1312G > A, p.Val438Met) was predicted to be deleterious using SIFT; probably damaging using PolyPhen; and disease causing using MutationTaster. However, the novel risk allele (G) was predicted to create a binding site for a miRNA (hsa-miR-377-3p) that is also predicted to regulate genes involved in neuronal metabolic and oxidative stress [34]. The novel SNP rs255114 (MDD/T2D-comorbid risk) is located in a DNAase-hypersensitive site and a TF-binding site [32]. The novel risk allele (C) of SNP rs1003929 (MDD-risk) was predicted to affect the histone modification (H3K27me3) in neural tissue, causing an epigenetic modification of histone H3 associated with gene down-regulation [35] (Figure 5).

## 3. Discussion

Depression and T2D are both associated with high morbidity and impaired quality of life [36]. They share many etiological mechanisms and intersecting pathways, including the HPA-axis [37] and insulin resistance [38]. Our novel findings demonstrate that in families with T2D, the *CRHR2* gene contributes to both disorders via pleiotropism and underlies the shared genetic pathogenesis of their comorbidity. Within *CRHR2*, we detected five novel unique SNPs that were significantly linked to or in LD (i.e., association) with MDD; five novel unique SNPs that were linked to or in LD (i.e., association) with T2D; and two novel SNPs that were linked to both diseases across different modes of inheritance. All detected variants are novel both in MDD and T2D. One variant (rs7812133) has been previously studied in 66 MDD cases and 29 control subjects in relation to cortisol level and severity of depression [25]; however, the association was statistically non-significant, possibly due to the limited study size. In our study, the novel rs7812133 variant was significantly found in linkage to and LD/association with T2D (*p* = 0.04), and, as reported in the previous study [25], does not contribute to depression. The novel T2D-risk rs8192498 variant detected within our study was previously found to be associated with resting metabolic rate and energy expenditure [39], but was not found to be associated with suicide in traumatized children [40] or panic disorder [41], consistent with our negative finding in depression. The SNP rs77113016 identified to be a novel T2D-risk variant in our familial dataset was previously found in a severely obese child with hyperphagia, but it did not segregate with obesity in other family members [42].

Interestingly, the variants linked to or in LD (i.e., association) with MDD had generally higher statistical significance level than the variants linked to or in LD (i.e., association) with T2D, despite the fact that the families were primarily ascertained for T2D, indicating that *CRHR2* variants may confer a risk for MDD more strongly than for T2D. However, the most significant SNPs detected are either MDD-risk or T2D-risk variants. Of note, some of the MDD-risk variants were found within a novel LD block (Set01) which was not shared by the T2D-risk variants. The novel Set01 specific LD block related to MDD allows us to infer that a specific region in *CRHR2* gene is linked to MDD and that the presence of other independent variants may confer additional risk for MDD and/or T2D. These results align with the concept that sequential stressors or chronic stress in the setting of genetic predisposition to HPA-axis hyperactivation may maintain chronic hypercortisolemia and lead primarily to depression [43] via the mediated serotonin down-regulation [44] and secondarily to T2D potentially via the long-term effects of chronic hypercortisolemia. In fact, *CRHR2-*knockout in mice led to stress hyper-responsiveness [17], and *CRHR2*-blockade in rats abolished stress-induced neuronal serotonin synthesis [45]. In addition, *CRHR2-*knockout mice developed high blood pressure [46], increased feeding [46], and impaired glucose tolerance [47], all traits related to T2D and metabolic syndrome [48]. In humans, the *CRHR2* locus 7p21-p15 has been linked to depression [19], bipolar disorder [49,50], obesity [51], T2D [18], and increased HDL and triglyceride levels [52]. In addition, CRHR2 antagonists attenuated the effect of brain-derived neurotrophic factor (BDNF) on reducing food intake and body weight [53]. Among previous human studies, the association of *CRHR2* gene variants with MDD was not consistent. A rare *CRHR2* variant (rs3779250-risk allele-C) has been reported in Japanese patients with MDD [21], and a previous study in Belgium reported an MDD-risk variant with borderline significance (*p* = 0.04) [54]. However, a later study in Denmark [55] reported no positive findings, probably due to ethnic differences. In addition, epigenetic changes in the *CRHR2* gene were associated with MDD in female adolescents [56]. *CRHR2* variants have also been reported in patients with bipolar disorder (rs8192492-risk allele-A) [22], PTSD (rs2267715-risk allele-A) [23], and T2D (rs917195 T2D-risk allele-C) [24]; and certain haplotypes have been associated with suicidal risk in bipolar patients [57].

In our study, the novel SNPs rs2284220, rs1003929, and rs117157639 in LD/association with MDD, novel rs7812133 in LD/association with T2D, and novel rs255114 in LD/association with both disorders are likely the most relevant in the disorders’ pathogenesis. Large-scale case–control studies are needed to replicate these findings. However, as rs2284220 and rs1003929 lie within the same LD block, each one is a potential replicate of the other. Further, the two novel independent MDD-T2D comorbid SNPs, rs10271601 and rs255114, might underlie the pleiotropic effects of *CRHR2* on mood and metabolic disturbances. The in silico functional analysis for the significantly linked SNPs predicted overall regulatory gene down-regulation (rs2284220, rs255114, rs1003929, rs117157639, rs77113016, and rs7812133) and perhaps lower CRHR2 density. *CRHR2* down-regulation may be associated with the same effects seen in the *CRHR2*-knockout mice which manifested stressed and anxiety-like behavior [17]. *CRHR2* down-regulation may also lead to hypercortisolemia via an inappropriately high-normal CRH and ACTH, via CRHR1 pituitary activation, and simultaneously drive central and peripheral catecholamine secretion, which can further mediate the anxiety-causing and hyperglycemic effects [58].

## 4. Methods and Materials

We used the dataset of 212 families descended from at least 3 generations of Caucasian Italians originating from the Italian peninsula. Both sexes were included. Families with identical twins and siblings with uncertain paternity were excluded. The families had familial T2D [59,60] and were phenotyped for the presence or absence of MDD using DSM-IV diagnostic criteria [61]. All subjects and data were fully deidentified. Families were previously recruited in Italy, following the Helsinki declaration guidelines, and provided written informed consent prior to participation. The study was institutionally approved by the Jefferson Ethical Committee.

In the family subjects, we amplified 17 *CRHR2* single nucleotide polymorphisms (SNPs) by microarray. We performed genotyping and Mendelian error exclusion using PLINK [62]. Using Pseudomarker [63], we analyzed the 17 SNPs in *CRHR2* for 2-point parametric-linkage to and LD (i.e., association) with T2D and MDD using the following models: dominant with complete penetrance (D1), dominant with incomplete penetrance (D2), recessive with complete penetrance (R1), and recessive with incomplete penetrance (R2). Pseudomarker [63] uses statistical algorithms to perform parametric testing of both linkage and LD in affected and non-affected individuals. It provides 5 test statistics: Linkage, LD|Linkage, LD|NoLinkage, Linkage|LD, and LD + Linkage. The probability of an allele being linked or in LD to the phenotype is expressed in *p* values. In our study, we used (*p* ≤ 0.05) as cut off for statistical significance. To test the presence or absence of LD blocks within the variants showing statistically significant results in T2D or MDD (*p* ≤ 0.05), we computed LD correlations via LD matrix among the SNPs available in the Toscani Italian population from the 1000 Genomes Project (https://www.internationalgenome.org/data-portal/population/TSI) (accessed on 28 September 2021) [64]. The SNPs that significantly correlated (r^2^ ≥ 0.9) with other SNPs were considered within the same LD block and labeled based on that unique LD block (e.g., Set 01, Set 02). All SNPs designated as “Independent” were not correlated with any other SNPs.

## 5. Conclusions

This is the first study detecting *CRHR2* gene pleiotropism underlying genetic comorbidity of MDD and T2D in T2D families. The relatively higher genetic homogeneity of the Italian families derived from a peninsula-based population may have increased the detection power of our analysis [65]. However, this study has limitations. The relationship between the *CRHR2* SNPs and the metabolic disturbances and MDD can be indirect if the SNP is in linkage but not in LD, that is association, with the phenotype, and thus it is not in LD with the putative risk SNP. Some of the SNPs we detected were in LD with T2D and/or MDD as well as both in linkage and LD with T2D and/or MDD. However, to prove which SNP risk allele produces biological effects, in vitro studies are need. In fact, the SNP in LD with MDD and/or T2D might be in LD with another SNP which is the one having the major biological effects. Disentangling biological effects from risk SNPs in LD is not trivial. The predicted functional variants reported in our study should be experimentally validated; hence functional studies are needed. Our study is limited to one homogeneous ethnic group; therefore, the findings should be replicated in other ethnicities in order to draw a better picture of the role of the *CRHR2* gene in these complex diseases.

## Figures and Tables

**Table 1:** *CRHR2*-Risk Single Nucleotide Polymorphisms (SNPs) for Major Depressive Disorder (MDD) and Type 2 Diabetes (T2D).

Disease	Model ^1^	SNP	Position	Ref	Alt	Risk Allele	Consequence	LD Block	Reported in MDD or T2D
MDD	D1, D2, R2	rs2284220	chr7:30678487	G	A	G	Intronic	Set01	Novel
	D2, R2	rs1003929	chr7:30679433	T	C	C	Intronic	Set01	Novel
	R1	rs117157639	chr7:30688084	C	G	C	Intronic	Independent	Novel
	D1, R1, R2	rs10271601	chr7:30692377	C	T	T	Intronic	Independent	Novel
	D1, R1	rs255114	chr7:30698941	C	T	C	Intronic	Independent	Novel
T2D	D1, D2	rs77113016	chr7:30653465	C	T	C	Missense	-	Novel
	D1, R1	rs7812133	chr7:30655790	G	A	G	Intronic	Independent	Studied with MDD but no association [25]
	D2	rs8192498	chr7:30662196	C	T	T	Missense	Independent	Novel
	D1	rs10271601	chr7:30692377	C	T	T	Intronic	Independent	Novel
	R1, R2	rs255114	chr7:30698941	C	T	C	Intronic	Independent	Novel

Risk SNPs for MDD and T2D are shown, with indication of the significant inheritance model(s), the chromosomal base pair position, the risk allele, the gene site or consequence of the variant, the indication whether the single nucleotide polymorphisms (SNPs) lies in linkage-disequilibrium block or is independent, and the indication whether the SNP was previously detected in MDD or T2D. ^1^ Models: D1: dominant, complete penetrance; D2: dominant, incomplete penetrance; R1: recessive, complete penetrance; R2: recessive, incomplete penetrance.

## Data Availability

The data presented in this study are available on reasonable request. The data are not publicly available due to privacy restrictions, lacking specific patients’ consent.

## Abbreviations

ACTH	adrenocorticotropin-releasing hormone
CRH	corticotropin-releasing hormone
D1	dominant with complete penetrance
D2	dominant with incomplete penetrance
HPA	hypothalamic–pituitary–adrenal axis
LD	linkage disequilibrium
MDD	Major Depressive Disorder
PTSD	post-traumatic stress disorder
R1	recessive with complete penetrance
R2	recessive with incomplete penetrance
SNPs	single nucleotide polymorphisms
T2D	type 2 diabetes
